# Reconstruction and Analysis of the lncRNA-miRNA-mRNA Network Based on Competitive Endogenous RNA Reveal Functional lncRNAs in Dilated Cardiomyopathy

**DOI:** 10.3389/fgene.2019.01149

**Published:** 2019-11-15

**Authors:** Lichan Tao, Ling Yang, Xiaoli Huang, Fei Hua, Xiaoyu Yang

**Affiliations:** ^1^Department of Cardiology, The Third Affiliated Hospital of Soochow University, Changzhou, China; ^2^Department of Endocrinology, The Third Affiliated Hospital of Soochow University, Changzhou, China

**Keywords:** long non-coding RNA, microRNA, competitive endogenous RNA, lncRNA-miRNA-mRNA network, dilated cardiomyopathy

## Abstract

Dilated cardiomyopathy (DCM) is an important cause of sudden death and heart failure with an unknown etiology. Recent studies have suggested that long non-coding RNA (lncRNA) can interact with microRNA (miRNA) and indirectly interact with mRNA through competitive endogenous RNA (ceRNA) activities. However, the mechanism of ceRNA in DCM remains unclear. In this study, a miRNA array was first performed using heart samples from DCM patients and healthy controls. For further validation, we conducted real-time quantitative reverse transcription (RT)-PCR using samples from DCM patients and a doxorubicin-induced rodent model of cardiomyopathy, revealing that miR-144-3p and miR-451a were down-regulated, and miR-21-5p was up-regulated. Based on the ceRNA theory, we constructed a global triple network using data from the National Center for Biotechnology Information Gene Expression Omnibus (NCBI-GEO) and our miRNA array. The lncRNA-miRNA-mRNA network comprised 22 lncRNA nodes, 32 mRNA nodes, and 11 miRNA nodes. Hub nodes and the number of relationship pairs were then analyzed, and the results showed that two lncRNAs (NONHSAT001691 and NONHSAT006358) targeting miR-144/451 were highly related to DCM. Then, cluster module and random walk with restart for the ceRNA network were analyzed and identified four lncRNAs (NONHSAT026953/NONHSAT006250/NONHSAT133928/NONHSAT041662) targeting miR-21 that were significantly related to DCM. This study provides a new strategy for research on DCM or other diseases. Furthermore, lncRNA-miRNA pairs may be regarded as candidate diagnostic biomarkers or potential therapeutic targets of DCM.

## Introduction

Chronic heart failure (CHF), a main cause of morbidity and mortality, especially in aging. CHF is a complex clinical syndrome resulting from various structural and functional impairments in ventricular filling or blood ejection (Garin et al., 2014). The lifetime risk of developing CHF has been calculated to range from 20 to 33% worldwide, and nearly half of the patients with CHF will die within 5 years despite all the advanced therapies (Dobre et al., 2014). In addition, as the population ages, the cost associated with CHF is also expected to increase substantially. The etiology of CHF can be classified as ischemic (ICM) or non-ischemic cardiomyopathy (NICM), and dilated cardiomyopathy (DCM) is one of the major causes of ICM. In contrast to revascularization therapies for ICM, novel treatments for DCM remain scarce. Therefore, studies focused on developing new strategies for DCM are urgently required.

Accumulating evidence has suggested that rather than being transcriptional noise, diverse non-coding RNAs (ncRNAs) serve as master regulators in CHF initiation and progression at the post-transcriptional level (Kumarswamy and Thum, 2013; Pinet and Bauters, 2015; Thum, 2015). Among them, long non-coding RNAs (lncRNAs) are conventionally described as transcripts longer than 200 nucleotides with no or little protein-coding capacity (Greco et al., 2016; Dangwal et al., 2017). Owing to their versatility, lncRNAs have been reported to participate in several cellular processes ranging from chromatin modification and RNA stability to translational control. Biochemically, lncRNAs exert their function *via* RNA-RNA, RNA-DNA, or RNA-protein interactions (Li et al., 2013; Dey et al., 2014; Shi et al., 2015). Of note, lncRNAs have been reported to competitively interact with microRNAs (miRNAs) and thus inhibit target mRNA degradation by a competitive endogenous RNA (ceRNA) regulatory mechanism (Sen et al., 2014; Tay et al., 2014).

Recently, studies have identified several aberrantly expressed lncRNAs in CHF models. Moreover, overexpression/knockdown of specific lncRNAs have been reported to significantly influence the process of cardiac development, aging, hypertrophy, and fibrosis in mice (Cheng et al., 2014; Michalik et al., 2014; Devaux et al., 2015; Pourrajab et al., 2015; Uchida and Dimmeler, 2015). However, because of low sequence conservation among species, it is difficult to extend the findings derived from murine models to humans; therefore, little is known about the function of lncRNAs in human hearts. Current reports of lncRNAs in DCM patients are focused on expression profiles from RNA sequencing or microarray (Schiano et al., 2017; Haas et al., 2018). Therefore, considering the large number and limited knowledge of lncRNAs, how to develop computational model for identification of lncRNAs and downstream miRNAs or mRNAs are of significant importance.

In our study, we conducted a microarray profile of miRNAs in myocardial biopsy samples from end-stage DCM patients compared with those in normal myocardial samples. Furthermore, based on the ceRNA theory, we constructed a global triple network by using data from the GEO database, as lncRNA and mRNA form a triplet by sharing the same miRNA. We identified human DCM-related lncRNAs with high reliability and our results showed that the lncRNA-miRNA-mRNA network provides a new understanding of the mechanisms and potential therapeutic targets for DCM.

## Materials and Methods

### Patients and Tissue Samples

**Figure 1:**
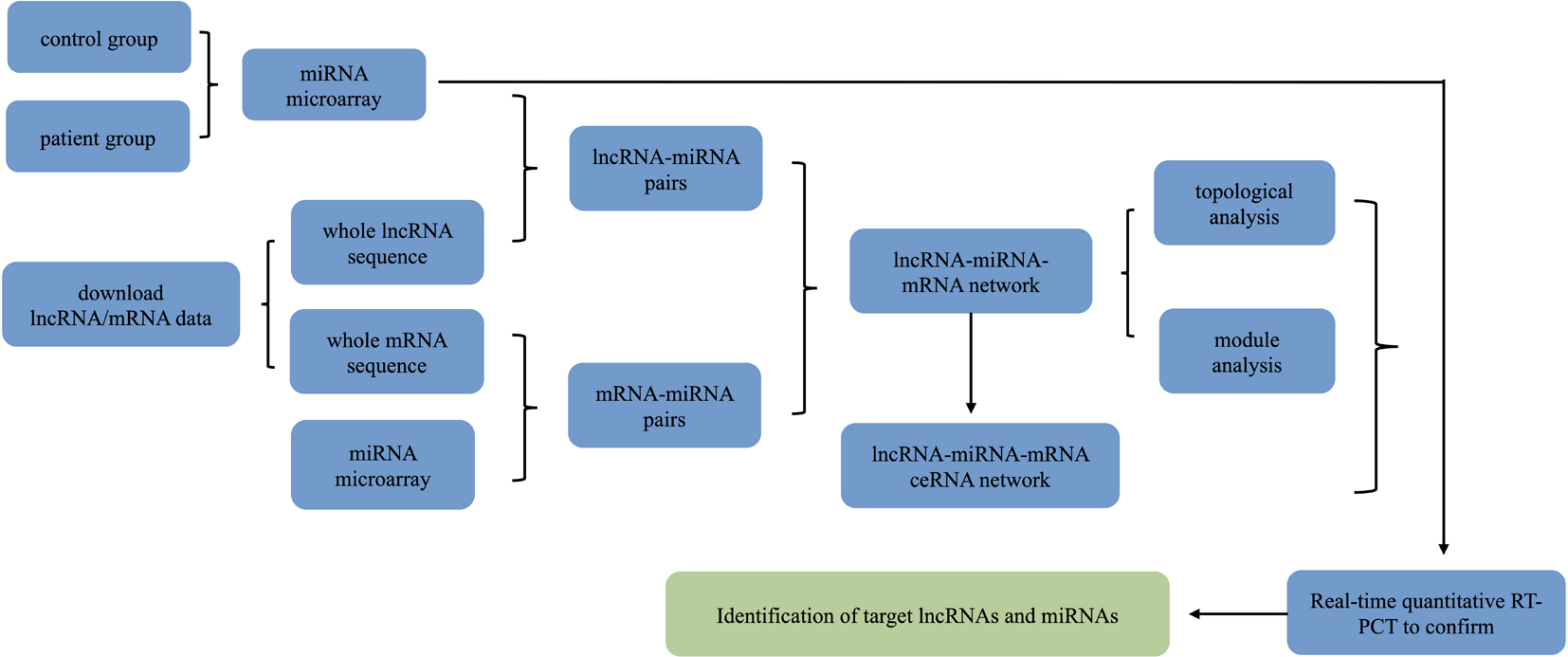
Schematic depiction of the experimental design and flowchart of the steps performed in this study.

The experimental procedure for evaluating the differential expression of lncRNA/miRNA/mRNA is described in (**Figure 1**) The study protocol was approved by the Medical Ethics Committee of the Third Affiliated Hospital of Soochow University in Changzhou, Jiangsu Province, China, and informed consent was obtained from each patient. Tissues for detection were collected from the left ventricular wall of explanted hearts from patients with a diagnosis of DCM undergoing heart transplantation (clinical data were presented in our previous paper) (Tao et al., 2016) and from unmatched healthy donors.

### Animal Model

Doxorubicin-induced cardiomyopathy mouse model was induced by chronically administrating mice with either doxorubicin or phosphate-buffered saline (PBS) by six intraperitoneal (i.p) injections (day 0, 2, 4, 6, 8, and 10) at a dose of 4 mg/kg. After 4 weeks, echocardiography was performed and mice were sacrificed.

### RNA Isolation

Total RNA was harvested using TRIzol and purified with the RNeasy mini kit (Qiagen, Hilden, Germany) according to manufacturer’s instructions. cDNA synthesis was performed with Bio-Rad iScripTM cDNA Synthesis Kit (Bio-Rad, Hercules, CA, USA) in a reaction volume of 10 µl.

### miRNA Microarray Gene Expression Profiling

The miRNA expression profiling assay system based on Affymetrix 4.0 (OE Biotech’s, Shanghai, China) was used to perform miRNA expression profiling of myocardial samples from three patients with DCM and three healthy donors (No. GSE112556). Clinical and echocadiography parmeters for patients with dilated cardiomyopathy were presented in **Supplemental Table 1**. The threshold of up-regulated or down-regulated miRNA was a fold change greater than two, and *P* < 0.05 using Student’s t-test was considered statistically significant.

### lncRNA and mRNA Microarray Data

GEO is a public functional genomics data repository that supports MIAME-compliant data submissions. Human lncRNA/mRNA expression profiles were downloaded from NCBI-GEO (GSE42955) (Molina-Navarro et al., 2013). The threshold of up-regulated or down-regulated lncRNA/mRNA was a fold change greater than 1.5, and *P* < 0.05 using Student’s t-test was considered statistically significant. This database was further analyzed with our miRNA microarray profile.

### Real-Time Quantitative Reverse Transcription (Rt)-PCR

For quantitative mRNA analysis, a template equivalent to 400 ng of total RNA was subjected to 40 cycles of quantitative PCR using the Takara SYBR Premix Ex TaqTM (TliRNaseH Plus, Takara, Tokyo, Japan) in the 7900HT Fast Real-Time PCR System. The absolute expression levels of miRNAs were normalized to the internal control small nuclear U6 and the expression levels of lncRNAs were normalized to 18s, all the data were calculated by the ΔΔCt method. Each reaction was primed using a gene-specific stem-loop primer. The RT stem-loop primers and PCR primers are listed in **Supplemental Table 2**.

### Prediction of the miRNA Targets of lncRNAs and mRNAs

The miRNA targets of lncRNA were predicted and the minimum free energy (MFE) of miRNA-lncRNA duplexes was calculated by using the RNAhybrid program. MiRNA sequences were obtained from miRbase and lncRNA sequences were obtained from NCBI-nucleotide. MiRNA target binding sites on the entire lncRNA sequence were predicted. Data of miRNA-mRNA interactions were downloaded from the Miranda and Targetscan prediction tools.

### Construction of the lncRNA-miRNA-mRNA Network

The lncRNA-miRNA-mRNA network was constructed based on ceRNA theory as follows: (1) Expression correlation between lncRNAs and mRNAs was calculated using the Pearson correlation coefficient (PCC). The lncRNA-mRNA pairs with PCC > 0.99 and *P* value < 0.05 were selected as target pairs. (2) Among the selected lncRNA-mRNA pairs, if both the lncRNA and mRNA targeted and were negatively co-expressed with a common miRNA, this lncRNA-miRNA-mRNA group was identified as a co-expression competing triplet. (3) The lncRNA-miRNA-mRNA network was visualized by using Cytoscape software.

### Functional Enrichment Analysis

Significant progress in data mining has provided a wide range of bioinformatics analysis tools, including the gene ontology (GO) and Kyoto Encyclopedia of Genes and Genomes (KEGG) databases. The GO database provides gene ontologies, the annotations of genes, and gene products to terms. The combination of ontologies and annotations renders the GO database as a powerful tool for further analysis. The KEGG database is a relational database comprising searchable molecular interaction pathways and reaction networks in metabolism, various cellular processes, and multiple human diseases.

### Reconstruction of the Key lncRNA-miRNA-mRNA Subnetwork

Every lncRNA and its related miRNAs and mRNAs in the global triple network were extracted to construct a new subnetwork using Cytoscape software. The number of related lncRNA-miRNA-mRNA triplets was calculated. By comparing the node degree of lncRNA and the number of related lncRNA-miRNA and miRNA-mRNA pairs, the target lncRNAs were identified. We then performed qRT-PCR to confirm the changes of lncRNA in samples from DCM patients and healthy controls. For further analysis, GO and KEGG enrichment analyses were performed for each of the validated lncRNAs by using their mRNA neighbors in the lncRNA-miRNA-mRNA subnetwork.

### Neonatal Rat Ventricular Cardiomyocyte and Fibroblast Isolation and miRNA Transfection

All rats were purchased and raised in the Experimental Animal Center of Soochow University (Suzhou, Jiangsu Province, China). All procedures were in accordance with guidelines on the use and care of laboratory animals for biomedical research published by the National Institutes of Health (No. 85–23, revised 1996), and the experimental protocol was approved by the Animal Care and Use Committee of Soochow University. The isolation of neonatal rat ventricular cardiomyocytes (NRCM), fibroblasts (NRCF), and miRNA transfection were conducted as described in our previous paper (Tao et al., 2018).

All transfection and assays on cardiomyocytes and fibroblasts were conducted in serum free medium and low serum medium (1% FBS), respectively. Cardiac fibroblasts at passage two were exposed to miRNA agomir versus negative control (50 nM), or antagomir versus negative control (100 nM) (RiboBio, Guangzhou, China) for 48 h, and treated with 5 ng/ml or 10 ng/ml TGFβ (Peprotech, Rocky Hill, NJ, USA) for 24 h.

### Statistical Analysis

In this study, data are expressed as the mean ± SD. Student’s t-test, Chi-square test, or one-way ANOVA followed by Bonferroni’s *post hoc* test was used to compare the one-way layout data when appropriate. *P* values less than 0.05 were considered to be significantly different. All analyses were performed using GraphPad Prism 5.

## Results

### Screening for Differentially Expressed lncRNAs, miRNAs, and mRNAs

**Figure 2:**
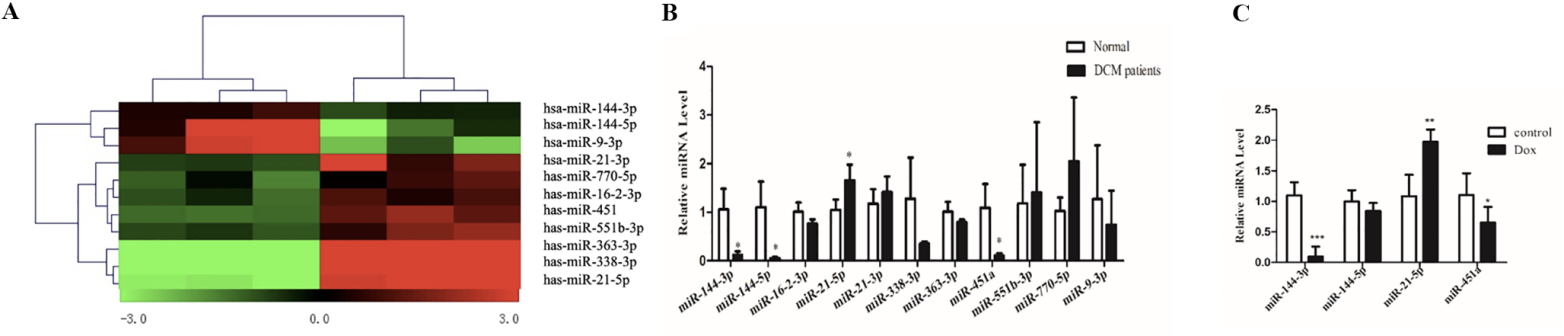
MiRNA array using heart samples from DCM patients and healthy controls. **(A)**. Heatmap of miRNA array between DCM patients and healthy controls and a total of 11 miRNAs were found to be dysregulated. **(B)**. qRT-PCR analysis of miRNA between DCM patients and healthy controls (n = 3). **(C)**. qRT-PCR analysis of miRNA between doxorubicin-induced DCM models and controls (n = 6). *, *p* < 0.05; **, *p* < 0.01; ***, *p* < 0.001.

miRNA arrays were used to determine differentially expressed miRNAs (DEMis) in samples from DCM patients and healthy controls (Owing to the difficulty of obtaining human heart tissues, the DCM sample size was small). A total of 11 miRNAs were found to be dysregulated (fold change > 2.0; *P* < 0.05, **Figure 2A** and **Table 1**). Based on the qRT-PCR analysis, we confirmed that miR-144-3p, miR-144-5p, and miR-451a were down-regulated and miR-21-5p was up-regulated in DCM heart samples (**Figure 2B**). To further confirm the changes in miRNA expression in DCM models, we determined the expression levels of miR-144-3p/5p, miR-451a, and miR-21-5p in a doxorubicin-induced cardiomyopathy rodent model (the echocardiographic parameters for the doxorubicin-induced DCM model were presented in **Supplemental Figure 1**). Interestingly, miR-144-3p and miR-451a were consistently down-regulated, and miR-21-5p was up-regulated in the DCM rodent model (**Figure 2C**).

**Table 1:** miRNA microarray in samples of DCM patients and healthy controls.

systematic_name	*P* value	Fold change	Regulation
hsa-miR-144-3p	2.57E-05	212.8677	down
hsa-miR-144-5p	3.18E-06	111.2795	down
hsa-miR-16-2-3p	0.047548	8.311692	down
hsa-miR-21-3p	0.032982	22.15763	Up
hsa-miR-21-5p	0.007706	2.248547	Up
hsa-miR-338-3p	0.030864	3.282057	down
hsa-miR-363-3p	0.006185	3.361872	down
hsa-miR-451a	3.08E-04	7.406941	down
hsa-miR-551b-3p	0.006586	5.052964	down
hsa-miR-770-5p	0.00854	20.36452	Up
hsa-miR-9-3p	1.55E-05	71.27749	down

Human lncRNA/mRNA expression data were obtained from the GEO database (GSE42955). Considering the differences in expression level between samples, the threshold of up-regulated or down-regulated lncRNAs/mRNAs was a fold change greater than 1.5, and *P* < 0.05 using Student’s t-test was regarded as statistically significant. A total of 61 lncRNAs and 172 mRNAs were selected for the following analysis.

### lncRNA-miRNA-mRNA ceRNA Network

**Figure 3:**
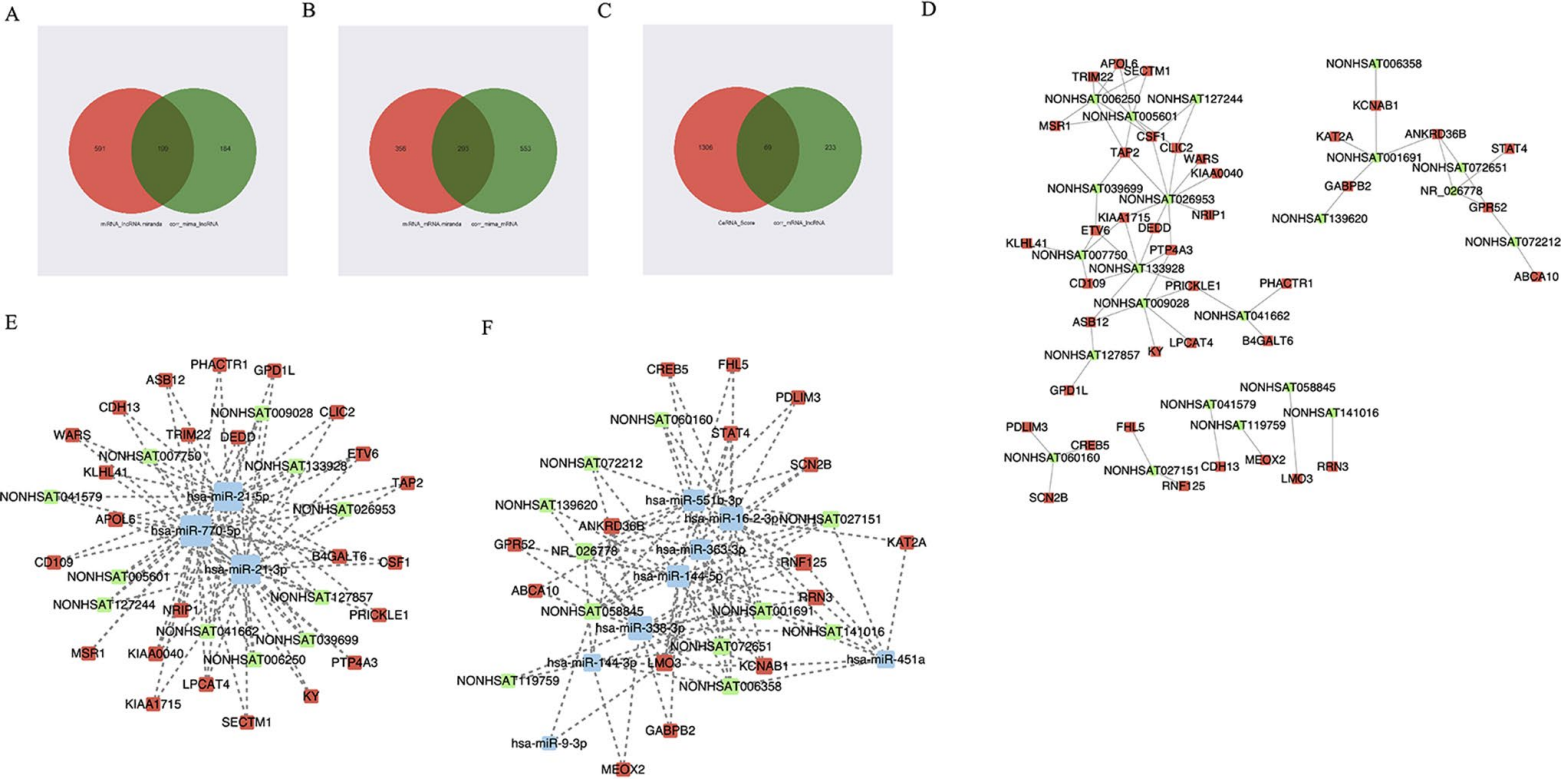
The view of lncRNA-miRNA-mRNA network. **(A–C)** 199 miRNA-lncRNA pairs and 293 miRNA-mRNA pairs were identified according to both base sequence and expression level. 69 lncRNA-mRNA pairs were selected according to the ceRNA score and expression level. **(D)** The view of lncRNA-mRNA network. The square represents mRNA and the circle represents lncRNA. **(E, F)** The view of lncRNA-miRNA-mRNA network. The red represents mRNA, the green represents lncRNA, and the blue represents miRNA.

First, we predicted lncRNA-miRNA and miRNA-mRNA pairs according to both base sequence and expression level. Considering that one miRNA may associate with several mRNAs or lncRNAs and that one lncRNA may also target several miRNAs, we analyzed whole miRNA microarray profiling. Based on the intersection elements, 199 miRNA-lncRNA pairs and 293 miRNA-mRNA pairs were identified (**Figures 3A, B**). Furthermore, 69 lncRNA-mRNA pairs were selected according to the ceRNA score and expression level (**Figures 3C, D**). Then the lncRNA-miRNA-mRNA network composed of 22 lncRNA nodes, 32 mRNA nodes, and 11 miRNA nodes were constructed (**Figures 3D–F**).

### Topological Analysis of the DCM-Related lncRNA-miRNA-mRNA Network

As we know, hub nodes play significant roles in biological networks. We first analyzed the topological properties of the DCM-related lncRNA-miRNA-mRNA network. We calculated the degree, closeness, and betweenness of the network, and we ranked all the node topological features of the network. We listed the top 20 of each dimension. Interestingly, we found that six lncRNAs (**Table 2**) appeared in the list. Moreover, the number of first relationship pairs of lncRNA-miRNA and secondary relationship pairs of miRNA-mRNA was calculated (**Table 3**). Among the top 11 lncRNA-miRNA pairs, seven lncRNAs were identified in the ceRNA network (NONHSAT072651, NONHSAT006358, NONHSAT001691, NONHSAT027151, NONHSAT072212, NONHSAT119759, and NONHSAT139620). It is worth noting that four lncRNAs (NONHSAT001691, NONHSAT072651, NONHSAT006358, and NONHSAT027151) not only had higher betweenness and node degree but also had a higher number of lncRNA-miRNA and miRNA-mRNA pairs, which suggested that these four lncRNAs may play crucial roles in the origin and development of DCM and may be selected as key lncRNAs.

**Table 2:** The top 20 genes in degree, betweenness and closeness.

Betweenness	Closeness	Degree	Pagerank	name	Gene type
248.5972516	0.028456914	31	0.088856743	hsa-miR-770-5p	miRNA
190.7634834	0.028388645	28	0.079988471	hsa-miR-21-3p	miRNA
174.639265	0.028365961	27	0.077025657	hsa-miR-21-5p	miRNA
125.3606094	0.025448029	19	0.042194577	hsa-miR-16-2-3p	miRNA
134.6788594	0.025448029	19	0.042922803	hsa-miR-338-3p	miRNA
72.16684166	0.025393419	16	0.035500316	hsa-miR-144-5p	miRNA
72.35483723	0.025375268	15	0.03428464	hsa-miR-551b-3p	miRNA
34.70901385	0.025339044	13	0.028012103	hsa-miR-363-3p	miRNA
17.53278481	0.025230988	8	0.018708332	hsa-miR-144-3p	miRNA
13.03038694	0.025230988	8	0.018263656	hsa-miR-451a	miRNA
18.6194442	0.025393419	7	0.015479801	NONHSAT001691	lncRNA
23.02997764	0.025375268	6	0.014139586	LMO3	mRNA
12.75746059	0.025375268	6	0.013537005	KCNAB1	mRNA
12.22486049	0.025375268	6	0.013492041	RNF125	mRNA
12.75746059	0.025375268	6	0.013537005	NONHSAT006358	lncRNA
12.22486049	0.025375268	6	0.013492041	NONHSAT027151	lncRNA
23.02997764	0.025375268	6	0.014139586	NONHSAT058845	lncRNA
12.95621164	0.025375268	6	0.013539288	NONHSAT072651	lncRNA
12.95621164	0.025375268	6	0.013539288	NR_026778	lncRNA
9.202070258	0.025357143	5	0.011653333	ANKRD36B	mRNA

**Table 3:** The number of lncRNA-miRNA and miRNA-mRNA pairs.

lncRNA	lncRNA–miRNA pairs	miRNA–mRNA pairs	Total number
NONHSAT077159	8	61	69
NONHSAT072651	8	61	69
NONHSAT033790	8	61	69
NONHSAT006358	8	61	69
NONHSAT001691	8	61	69
NONHSAT027151	8	61	69
NONHSAT072212	8	61	69
NONHSAT119759	8	61	69
NONHSAT139620	8	61	69
NONHSAT115202	8	61	69
NONHSAT018101	8	61	69

### Key lncRNA-miRNA-mRNA Subnetwork

**Figure 4:**
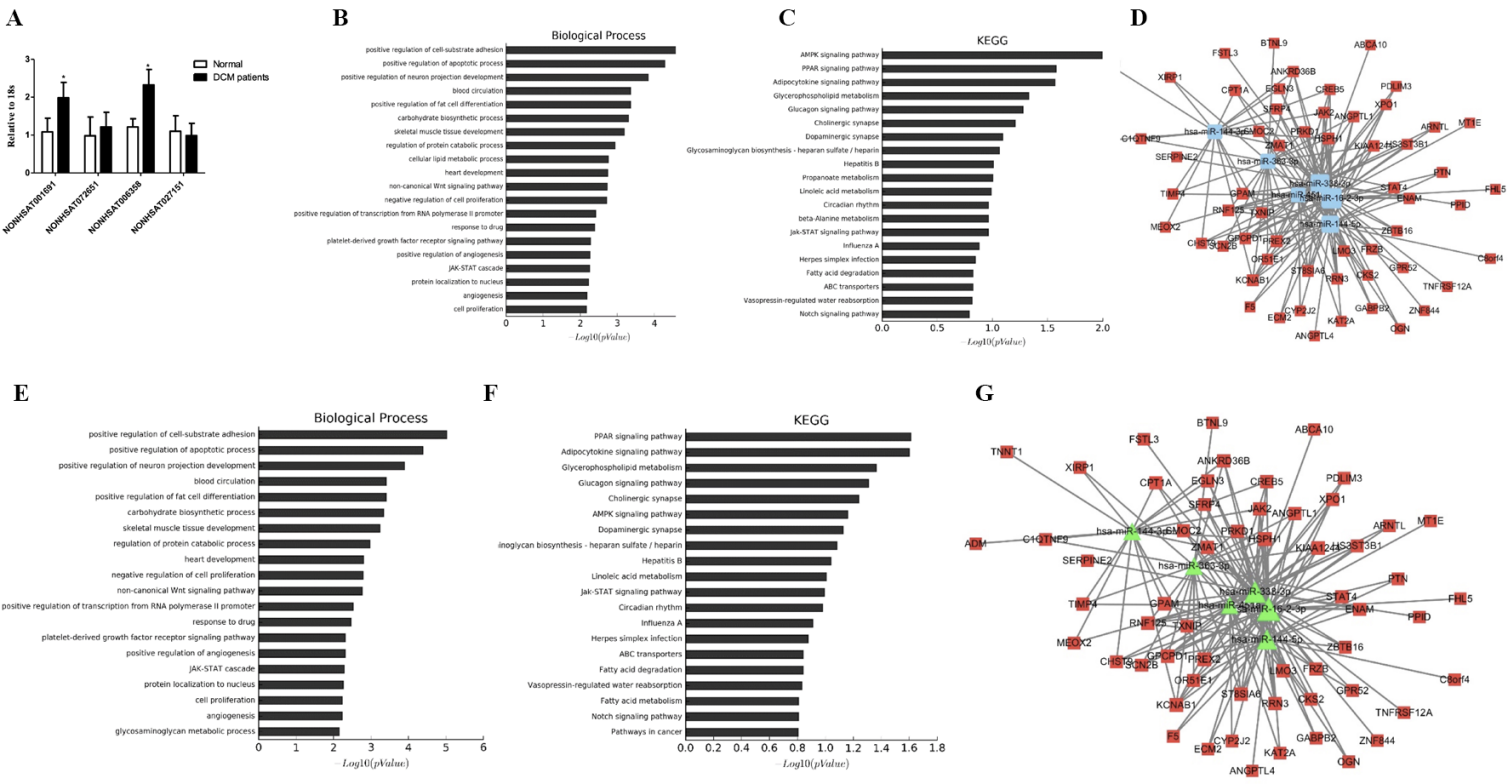
Key lncRNA-miRNA-mRNA subnetwork. **(A)**. qRT-PCR analysis of lncRNA between DCM patients and healthy controls (n-3). **(B, C)**. Biological function and pathway analysis of differentially expressed mRNAs related with NONHSAT001691. **(D)**. NONHSAT001691 related miRNA-mRNA subnetwork. The blue represents miRNA and the red represents mRNA. **(E, F)**. Biological function and pathway analysis of differentially expressed mRNAs related with NONHSAT006358. **(G)**. NONHSAT006358 related miRNA-mRNA subnetwork. The triangle represents miRNA and the square represents mRNA. *, *p* < 0.05.

We then searched for the four key lncRNAs in the ceRNA network we had previously constructed and found that the four lncRNAs mainly targeted miR-144-3p, miR-144-5p, and miR-451. To further validate the target lncRNA, qRT-PCR was performed using samples from DCM patients and healthy controls (primers for lncRNAs are presented in **Supplemental Table 2**). The results suggested that NONHSAT001691 and NONHSAT006358 were significantly increased in DCM patients (**Figure 4A**). We then identified the mRNA and miRNA associated with these two lncRNAs in the global triple network and reconstructed new subnetworks. GO function and KEGG pathway annotations for each of these two lncRNAs were performed. For NONHSAT001691, we identified the biological processes of “positive regulation of cell-substrate adhesion,” “positive regulation of apoptotic process” and “regulation of angiogenesis,” and the enriched KEGG pathways included the “AMPK signaling pathway,” PPAR signaling pathway,” and “adipocytokine signaling pathway” (**Figures 4B–D**). For NONHSAT006358, the biological processes were similar to those of NONHSAT001691, and the KEGG pathways included the “PPAR signaling pathway,” “adipocytokine signaling pathway,” and “glycerophospholipid metabolism” (**Figures 4E–G**). The subnetworks of lncRNAs NONHSAT001691 and NONHSAT006358 are presented in **Figures 4D, G**.

### Module Analysis of the DCM-Related lncRNA-miRNA Network

**Figure 5:**
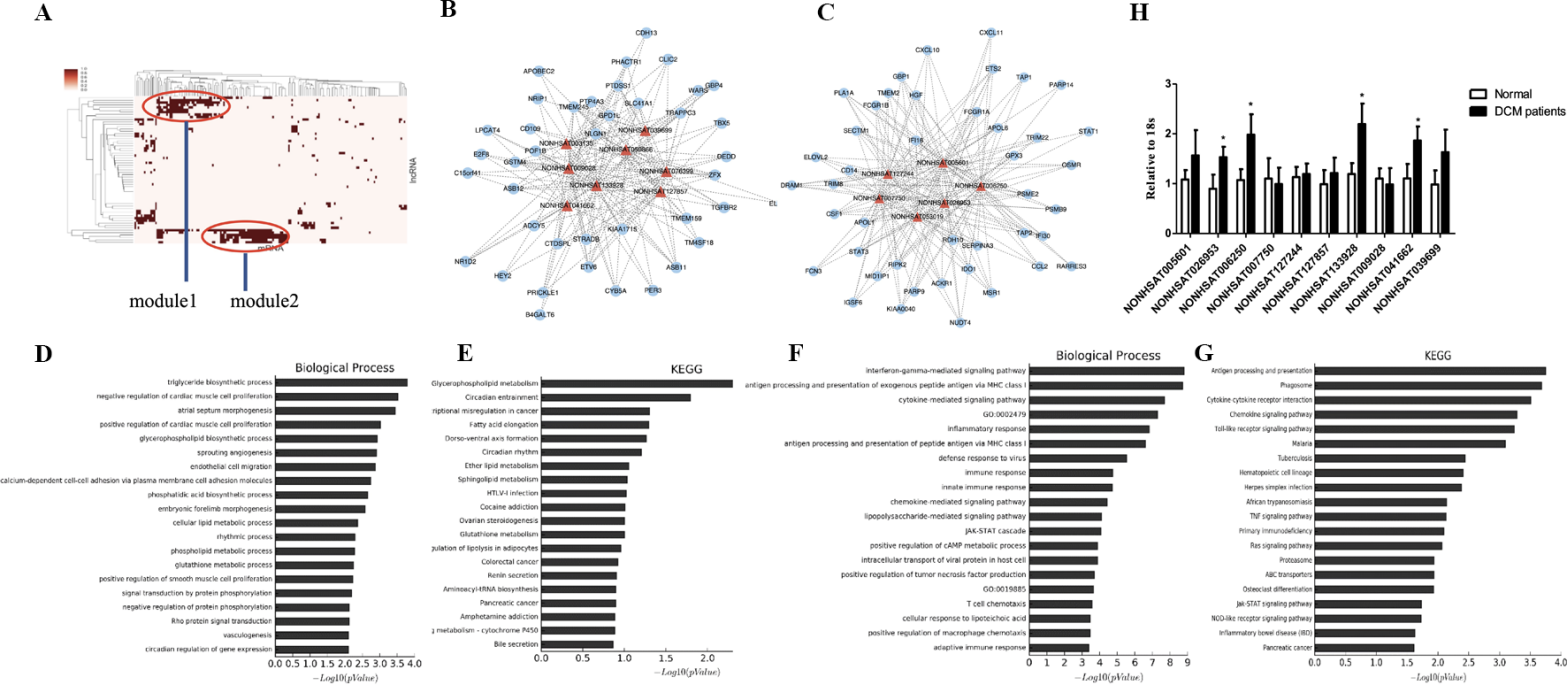
Module analysis of the DCM-related lncRNA-miRNA network. **(A)**. 2 modules were identified highly related to DCM. **(B, C)**. lncRNA-mRNA networks in 2 modules. The triangle represents lncRNA and the circle represents mRNA. **(D–G)**. Biological function and pathway analysis of differentially expressed mRNA related with 2 modules. **(H)**. qRT-PCR analysis of lncRNA between DCM patients and healthy controls (n = 3). *, *p* < 0.05.

To further investigate the crosstalk between mRNAs and lncRNAs, we performed bidirectional hierarchical clustering by using R package “gplot.” In the heat map, we discovered two modules (**Figures 5A–C**) that were highly related to DCM. Then, we performed GO enrichment analysis and KEGG analysis of genes in the modules (**Figures 5D–G**). In module 1, the “triglyceride biosynthetic process” was significantly and highly related to DCM. KEGG analysis demonstrated that “glycerophospholipid metabolism” was the most significant signaling pathway in DCM. In module 2, the “interferon gamma mediated signaling pathway” had the most notable relationship with DCM. Among all the lncRNAs in the two modules, we found 10 lncRNAs included in the ceRNA network (NONHSAT005601, NONHSAT026953, NONHSAT006250, NONHSAT007750, NONHSAT127244, NONHSAT127857, NONHSAT133928, NONHSAT009028, NONHSAT041662, and NONHSAT039699). Similar to previous approaches, we also conducted qRT-PCR using heart tissues from DCM patients and healthy controls. NONHSAT026953, NONHSAT006250, NONHSAT133928, and NONHSAT041662 were down-regulated in the DCM group. Interestingly, miR-21-5p was the major target of these four lncRNAs. As miR-21-5p has been validated in DCM patients, the involvement of these four lncRNAs in DCM was further confirmed.

### miR-144-3p/451a Play Different Roles in NRCM and NRCF *in Vitro*

**Figure 6:**
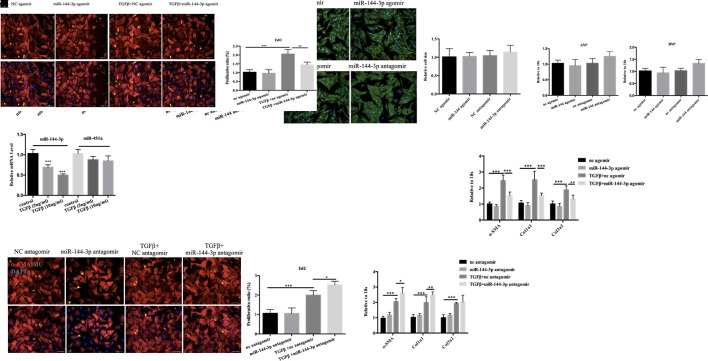
Functional study of miR-144-3p *in vitro*. **(A)**. qRT-PCR analysis of miRNA between cardiomyocytes and fibroblasts (n = 6). **(B, C)**. qRT-PCR analysis of the transfection efficacy of miR-144-3p with agomir/antagomir compared to controls (n = 6). **(C–E)**. Overexpression or downregulation of miR-144-3p did not perform effects on cardiomyocyte cell size and pathological hypertrophic markers (ANP and BNP), as evidence by α-actin/DAPI staining (n = 4) and qRT-PCR analysis (n = 6). **(F)**. Decreased expression of miR-144-3p in cardiac fibrosis model induced by TGFβ (n = 6). **(G, H)**. Forced expression of miR-144-3p attenuated TGFβ-induced cardiac fibroblast proliferation and trans-differentiation, as evidenced by EdU/α-SMA staining (n = 4) and qRT-PCR analysis of α-SMA, Col1a1 and Col3a1 (n = 6) **(I, J)**. inhibition of miR-144-3p deteriorated TGFb-induced cardiac fibroblasts proliferation and trans-differentiation as evidenced by EdU/α-SMA staining (n = 4) and qRT-PCR analysis (n = 6). Scale bar: 50um. *, *p* < 0.05; **, *p* < 0.01; ***, *p* < 0.001.

Next, we determined the relative expression level of miR-144/451a/21 in isolated neonatal rat cardiac cardiomyocytes versus fibroblasts and demonstrated higher expression level of miR-144-3p and miR-451a in cardiomyocytes, while miR-21-5p was enriched in fibroblasts compared to cardiomyocytes (**Figure 6A**). DCM is characterized by left ventricular dilation and interstitial fibrosis, which are the main causes of heart failure (McNally and Mestroni, 2017). MiR-21-5p was widely expressed in fibroblasts and has been reported to promote trans-differentiation from cardiac fibroblasts into myofibroblasts by targeting notch ligand Jagged1, contributing to cardiac fibrosis post myocardial infarction (Zhou et al., 2018). Then, we placed emphasis on miR-144-3p and miR-451a, which were closed clustered from a single gene locus. miR-451a has been reported to regulate cardiac hypertrophy and autophagy, forced expression of miR-451a in NRCM decreased cell size, whereas knockdown of miR-451a increased cell surface area (Song et al., 2014). Then we transfected miR-144-3p into NRCM and the transfection efficacy of miR-144 agomir or antagomir were exhibited in **Figure 6B**. However, overexpression or downregulation of miR-144 did not lead to any effects on cell size (**Figure 6C**) and markers of pathological hypertrophy (**Figures 6D, E**), supporting a more prominent role for miR-451a in cardiomyocyte hypertrophy rather than miR-144.

Cardiac fibrosis is another hall marker of DCM. By qRT-PCR, the expression level of miR-144-3p was identified downregulated in cultured neonatal rat cardiac fibrosis model stimulated by TGF-β while miR-451 did not show statistical significance (**Figure 6F**). To gain mechanistic insight into the role of miR-144 in regulating fibrosis, we investigated the effect of miR-144 in cardiac fibroblasts *in vitro*. MiR-144-3p overexpression decreased cardiac fibroblasts proliferation and trans-differentiation induced by TGF-β, as evidenced by a decrease in EdU and α-SMA staining, and decreased expression levels of α-SMA, Col1a1, and Col3a1(**Figures 6G, H**). Contrary to the effects of miR-144-3p overexpression, inhibition of miR-144-3p further enhanced cardiac fibroblasts proliferation and differentiation in the presence of TGF-β (**Figures 6I, J**), indicating a potential protective effect of miR-144-3p against cardiac fibrosis.

## Discussion

DCM is an important cause of sudden cardiac death (SCD) and heart failure and is the major indication of cardiac transplantation in children and adults worldwide. DCM is characterized by ventricular chamber enlargement and systolic dysfunction with excessive cardiac fibrosis (McNally and Mestroni, 2017). Over the past few years, great efforts have been made to explore the molecular mechanisms of DCM. miRNA-mediated myocardial gene expression is one of the novel mechanisms in DCM (Naga Prasad and Karnik, 2010; Miyamoto et al., 2015). In this study, we first performed a miRNA microarray using heart samples from DCM patients and healthy controls. miRNAs with a fold change > 2.0 and *P* value < 0.05 were further evaluated by qRT-PCR in DCM patients and a doxorubicin-induced cardiomyopathy rodent model. miR-144-3p and miR-451a were identified as down-regulated and miR-21-5p was identified as up-regulated in DCM.

Previous studies have demonstrated that miR-144 and miR-451 are closely clustered and evolutionarily conserved23. miR-144 and miR-451 are processed from a single gene locus that is regulated by the essential hematopoietic transcription factor GATA-4 (Zhang et al., 2010). miR-144/451 as a cluster have been identified to play crucial roles in cardiac ischemic lesions. In cardiomyocytes, ectopic expression of miR-144, or miR-451 augmented survival, which was further improved by overexpression of miR-144/451 cluster, compared to controls in response to simulated ischemia/reperfusion26. In a miR-144/451 knockout mouse model, loss of the miR-144/451 cluster limited the cardio-protection from ischemic preconditioning by up-regulating the Ras-related c3 botulinum toxin substrate 1 (Rac1)-mediated oxidative stress signaling pathway27, indicating a cardio-protective effects of miR-144/451a cluster against ischemic dysfunction. However, little studies have been done regarding the functional role of miR-144/451 on DCM-related pathological changes. Previous studies have shown that forced expression of miR-451a decreased cell size and knockdown of miR-451a performed opposite effects (Song et al., 2014). In our study, miR-144-3p did not show any effects on cell size on physiological conditions. Although miR-144-3p was enriched in cardiomyocytes compared to fibroblasts, overexpression of miR-144-3p attenuated fibroblast proliferation and trans-differentiation into myofibroblasts induced by TGF-β, which is also conforming with previous studies *in vivo* (He et al., 2018; Li et al., 2018). Therefore, although evolutionarily as a cluster, miR-144-3p and miR-451a may perform distinct roles in DCM. Furthermore, miR-144-3p may also participate in other pathological processes including inflammation (Hu et al., 2014), autophagy (Li et al., 2018), and mitochondrial metabolism (Li et al., 2016) on cardiomyocytes, which need to be explored in the future.

MiR-21 is one of the first identified miRNAs implicated in cardiac hypertrophy and fibrosis (Duygu and Da Costa Martins, 2015; Zhou et al., 2018). In our study, miR-21-5p was upregulated in DCM heart samples, which was consistent with larger sample study (Satoh et al., 2011). However, the regulatory effects of miR-21-5p on DCM were a result of single cardiac fibrosis or hypertrophy or a combination of diverse pathological process need further exploration.

In addition to miRNAs, accumulated data have shown that lncRNAs participate in a variety of biological processes and complex diseases, including DCM. Unfortunately, functional studies of lncRNAs are relatively more complicated than those of coding RNAs or miRNAs. Therefore, an efficient and accurate way to infer the potential function of lncRNAs is to detect their relationship with miRNAs and/or mRNAs, whose functions have been annotated. In our study, we used the interaction data from NCBI-GEO and our miRNA microarray to generate a global triple network based on the ceRNA theory, which suggests that lncRNAs and mRNAs share the same miRNA in one triplet. The lncRNA-miRNA-mRNA network was composed of 22 lncRNA nodes, 32 mRNA nodes, and 11 miRNA nodes. Then, the hub nodes and the number of relationship pairs were used to perform topological and subnetwork analysis. In general, a lncRNA with more relationship pairs indicates that the lncRNA is a hub that participates in more ceRNA interactions and plays essential roles in network organization. In this study, four lncRNAs (NONHSAT001691, NONHSAT072651, NONHSAT006358, and NONHSAT027151) were observed to be topological key nodes whose node degrees and number of lncRNA-miRNA and miRNA-mRNA pairs were significantly higher than other lncRNAs. These four lncRNAs were then validated in heart samples of DCM patients and healthy controls by using qRT-PCR. NONHSAT001691 and NONHSAT006358 were identified as significantly up-regulated in DCM patients. Interestingly, miR-144-3p and miR-451a were the potential targets of these two lncRNAs in the ceRNA network, indicating that the NONHSAT001691/NONHSAT006358-miR-144/451a signaling pathway may play a crucial role in the development of DCM.

GO and pathway analyses have been used to assess biological functions that are enriched among differentially expressed coding genes. Owing to similar miRNA targets, the significant GO terms of NONHSAT001691 and NONHSAT006358 shared common trends involving “positive regulation of cell-substrate adhesion and the apoptotic process,” and the results were consistent with those of previous studies on DCM (Miller et al., 2004; Pulinilkunnil et al., 2014; Isserlin et al., 2015). Pathway analysis of NONHSAT001691 and NONHSAT006358 showed that the metabolic pathways were mainly enriched, including the AMPK, PPAR, adipocytokine, glucagon, and fatty acid degradation signaling pathways, all of which have been shown to play important roles in DCM (Nikolaidis et al., 2004; Giannessi et al., 2011; Roh et al., 2014; Sung et al., 2015).

Moreover, bidirectional hierarchical clustering analysis was conducted to investigate the crosstalk between mRNAs and lncRNAs. A total of 10 lncRNAs were found in the ceRNA network, among which four lncRNAs (NONHSAT026953, NONHSAT006250, NONHSAT133928, and NONHSAT041662) were identified as down-regulated in the DCM group by qRT-PCR. miR-21-5p was the common target of these four lncRNAs, further confirming the feasibility of our miRNA microarray. Moreover, GO and KEGG analysis of these two modules also indicated that metabolism-related signaling pathways play a crucial role in the development of DCM, which provides a novel direction for the study of mechanisms underlying DCM.

Currently, discovering non-coding RNA-disease associations plays an increasingly vital role in devising diagnostic and therapeutic tools for diseases. However, since uncovering associations *via* experimental studies are expensive and time-consuming, novel and effective computational model for the identification of non-coding RNAs associated with DCM or other diseases are in demand. Several different computational methods have been analyzed to calculate potential non-coding RNA (including lncRNAs and miRNAs) -disease association scores (Chen and Yan, 2013; Chen and Huang, 2017; Chen et al., 2017; Chen et al., 2018a; Chen et al., 2018b; Chen et al., 2019). In our study, we constructed a lncRNA-miRNA-mRNA network based on the ceRNA theory. NONHSAT001691/NONHSAT006358-miR-144-3p/451a and NONHSAT026953/NONHSAT006250/NONHSAT133928/NONHSAT041662-miR-21-5p were further identified as potential key signaling pathways correlated with DCM. Therefore, this study provides the framework of constructing powerful computational model to predict potential lncRNA-miRNA-disease associations and select the most promising DCM or other disease-related lncRNAs/miRNAs for experimental validation. However, our study still has some limitations. First, owing to the difficulty of obtaining human heart tissues, the sample size was small, totaling six samples (six DCM samples and six healthy control samples). Due to the lack of samples, there may be false positives in the results. Second, in the process of converting different gene IDs from different databases, a number of genes may have been lost, which would decrease the accuracy of our results. Finally, our study was mainly focused on lncRNA/miRNA changes in DCM samples; therefore, the underlying biological functions need further exploration.

## Data Availability Statement

The data on miRNAs discussed in this manuscript have been deposited in NCBI’s GEO and are accessible through GEO Series accession number GSE112556.

## Ethics Statement

The study protocol was approved by the Medical Ethics Committee of the Third Affiliated Hospital of Soochow University in Changzhou, Jiangsu Province, China, and informed consent was obtained from each patient.

## Author Contributions

FH led the research team. LT conceived and designed the study.LY and XH developded the methodology. XY analyzed and interpreted the data. LT wrote, reviewed the manuscript.

## Funding

This work was supported by grants from the National Natural Science Foundation of China (grant no. 81700343), Natural Science Foundation of Jiangsu Province (grant no. BK20170296), China Postdoctoral Science Foundation (grant no. 2018M642317), Post-Doctoral Foundation of Jiangsu Province (grant no. 2018K095B), Six Talent Peaks Project of Jiangsu Province (grant no. WSN-202 to Lichan Tao, grant no. WSW-183 to Fei Hua), Maternal and Child Health Research Project of Jiangsu Province (grant no. F201803), and Changzhou High-Level Medical Talents Training Project (grant no. 2016ZCL J020).

## Conflict of Interest

The authors declare that the research was conducted in the absence of any commercial or financial relationships that could be construed as a potential conflict of interest.

## Supplementary Material

Supplemental Table 1 Clinical and echocardiography parameters for patients with dilated cardiomyopathy.

Supplemental Table 2 qRT-PCR Primers in this study.

Supplemental Figure 1 The echocardiographic parameters including ejection fraction (EF), fraction shortenting (FS), left ventricular mass, left ventricular internal dimension-systole (LVIDs) and left ventricular internal dimension-diastole (LVIDd) for the doxorubicin-induced DCM model. *, p < 0.05;**, p < 0.01; ***, p < 0.001.
