# Corrigendum: Reconstruction and Analysis of the lncRNA-miRNA-mRNA Network Based on Competitive Endogenous RNA Reveal Functional lncRNAs in Dilated Cardiomyopathy

**DOI:** 10.3389/fgene.2022.896402

**Published:** 2022-04-25

**Authors:** Lichan Tao, Ling Yang, Xiaoli Huang, Fei Hua, Xiaoyu Yang

**Affiliations:** ^1^ Department of Cardiology, The Third Affiliated Hospital of Soochow University, Changzhou, China; ^2^ Department of Endocrinology, The Third Affiliated Hospital of Soochow University, Changzhou, China

**Keywords:** long non-coding RNA, microRNA, competitive endogenous RNA, lncRNA-miRNA-, dilated cardiaomypothy

In the original article, an incorrect representative image of immunofluorescent staining for cardiac fibroblasts was contained in Figure 6G as published. The immunofluorescent staining of EdU/α-SMA in Figure 6G (nc agomir group) was duplicated as Figure 6I (nc antagomir group), which was mistakenly presented. The correct representative images for Figure 6G appear below.

**FIGURE 6:**
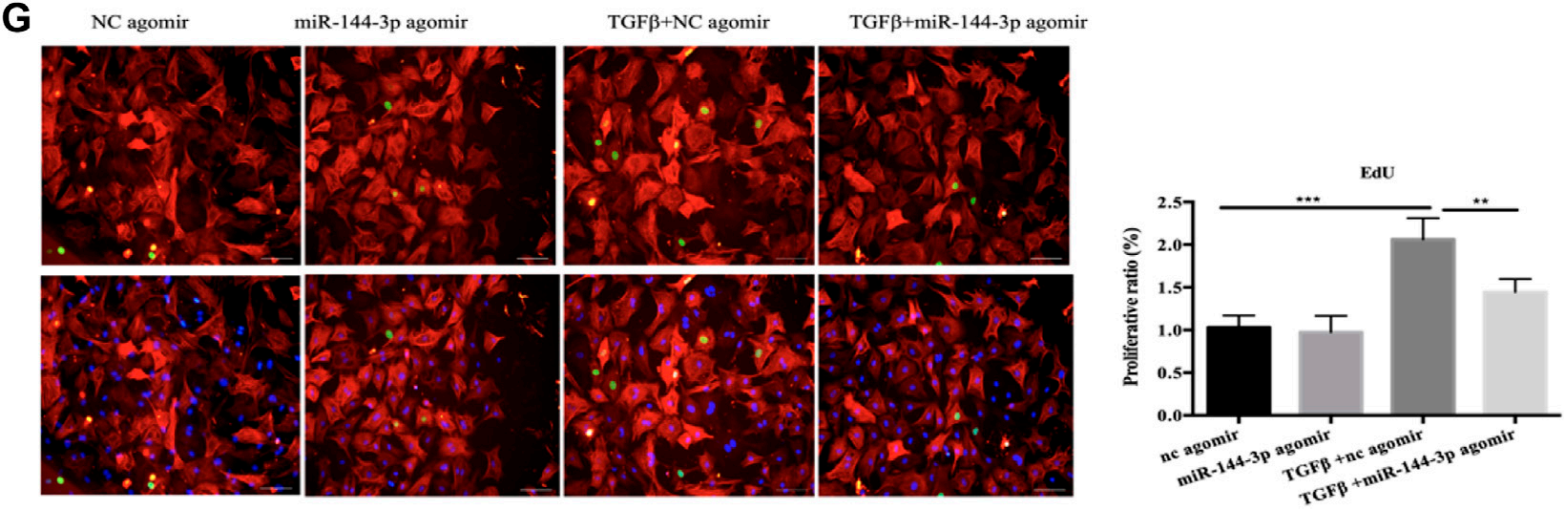
Functional study of miR-144-3p *in vitro*. **(A)**. qRT-PCR analysis of miRNA between cardiomyocytes and fibroblasts (n = 6). **(B,C)**. qRT-PCR analysis of the transfection efficacy of miR-144-3p with agomir/antagomir compared to controls (n = 6). **(C–E)**. Overexpression or downregulation of miR-144-3p did not perform effects on cardiomyocyte cell size and pathological hypertrophic markers (ANP and BNP), as evidence by α-actin/DAPI staining (n = 4) and qRT-PCR analysis (n = 6). **(F)**. Decreased expression of miR-144-3p in cardiac fibrosis model induced by TGFβ (n = 6). **(G,H)**. Forced expression of miR-144-3p attenuated TGFβ-induced cardiac fibroblast proliferation and trans-differentiation, as evidenced by EdU/α-SMA staining (n = 4) and qRT-PCR analysis of α-SMA, Col1a1 and Col3a1 (n = 6) **(I,J)**. inhibition of miR-144-3p deteriorated TGFb-induced cardiac fibroblasts proliferation and trans-differentiation as evidenced by EdU/α-SMA staining (n = 4) and qRT-PCR analysis (n = 6). Scale bar: 50um. *, *p* < 0.05; **, *p* < 0.01; ***, *p* < 0.001.

The authors apologize for this error and state that this does not change the scientific conclusions of the article in any way. The original article has been updated.

