# Supplementary material for: Reconstruction and Analysis of the lncRNA-miRNA-mRNA Network Based on Competitive Endogenous RNA Reveal Functional lncRNAs in Dilated Cardiomyopathy
Source: Front Genet. 2019 Nov 15;10:1149. doi: 10.3389/fgene.2019.01149 (PMC6873784; doi:10.3389/fgene.2019.01149)
Supplement: Supplemental Table 1 — Clinical and echocardiography parameters for patients with dilated cardiomyopathy. [file Table_1.docx]

Supplemental Table 1. Clinical and echocardiography parameters for patients with dilated cardiomyopathy

| parameters | mean±SD |
| --- | --- |
| Age (year) | 47.6±7.3 |
| LVEF (%) | 31.5±12.5 |
| LVFS (%) | 13.1±6.5 |
| LAD (mm) | 54.6±5.7 |
| LVDd (mm) | 75.4±14.5 |
| LVDs (mm) | 58.3±18.9 |
| IVS (mm) | 9.1±0.8 |
| LVPW (mm) | 8.7±0.9 |
| RAD (mm) | 49.3±7.4 |
| RVDd (mm) | 40.2±6.6 |

LVEF: left ventricle ejection fraction; LVFS: left ventricle fractional shortening; LAD: left atrium diameter; LVDd: left ventricular end diastolic dimension; LVDs: left ventricular end systolic dimension; IVS: interventricular septal thickness; LVPW: left ventricular posterior wall; RAD: right atrium diameter; RVDd: right ventricular end diastolic dimension.

Supplemental Table 2. qRT-PCR Primers in this study.

| Gene | Species | Forward | Reverse |
| --- | --- | --- | --- |
| α-SMA | rat | GTCCCAGACATCAGGGAGTAA | TCGGATACTTCAGCGTCAGGA |
| Col1a1 | rat | GAGCGGAGAGTACTGGATCGA | CTGACCTGTCTCCATGTTGCA |
| Col3a1 | rat | TGCCATTGCTGGAGTTGGA | GAAGACATGATCTCCTCAGTGTTGA |
| 18s | rat | AAGCTCACTGGCATGGCCTT | CGGCATGTCAGATCCACAAC |
| 18s | human | TGTGGGCATCAATGGATTTGG | ACACCATGTATTCCGGGTCAAT |
| NONHSAT001691 | human | GGAACCGAGCCCTAGAAACC | AAGCCCCACTTCGTCTTACG |
| NONHSAT072651 | human | ATGCAGTCGCAGTTAGCACT | TGCAAAGTTTACCTGGTGTGG |
| NONHSAT006358 | human | ACATACAGATGCAGTCCGCC | AGCCAGGTTTTCAGATTCAGGG |
| NONHSAT027151 | human | ACATACAGATGCAGTCCGCC | AGCCAGGTTTTCAGATTCAGGG |
| NONHSAT005601 | human | GAGGCTGGATGTTGCAAGGA | CCCTTGCCTGAGCAATGGTA |
| NONHSAT026953 | human | TCTACGTGGTGGAAACCCTG | ACGGTTCTGGAGACAATCCG |
| NONHSAT006250 | human | AGAGCTCTGAGGCTGGATGTT | CCTCAAGGCCAGAGGTTCTC |
| NONHSAT007750 | human | GCTTGGCTAGATTAGGGCCA | AATTGTAGACCAGTCCCCCTG |
| NONHSAT127244 | human | GCTTGGCTAGATTAGGGCCA | AATTGTAGACCAGTCCCCCTG |
| NONHSAT127857 | human | GAAAGAGTCCGCAAGGAGGT | ACAGGTTGCTTTCAGGCTGG |
| NONHSAT133928 | human | CAGCCTCATGTACTTCGCCT | CTAGCGTACTGTGAGACACCG |
| NONHSAT009028 | human | TAGCCCAGTGCCTAGTGTCT | CCTGACACTGGTGTTCCTCC |
| NONHSAT041662 | human | TAGCCCAGTGCCTAGTGTCT | CCTGACACTGGTGTTCCTCC |
| NONHSAT039699 | human | TCTCCTCGGGAAAAGCGAAC | GTAAGCGAGGGGAGTAAGGC |
