# Supplementary figures and images for: Reconstruction and Analysis of the lncRNA-miRNA-mRNA Network Based on Competitive Endogenous RNA Reveal Functional lncRNAs in Dilated Cardiomyopathy
Source: Front Genet. 2019 Nov 15;10:1149. doi: 10.3389/fgene.2019.01149 (PMC6873784; doi:10.3389/fgene.2019.01149)

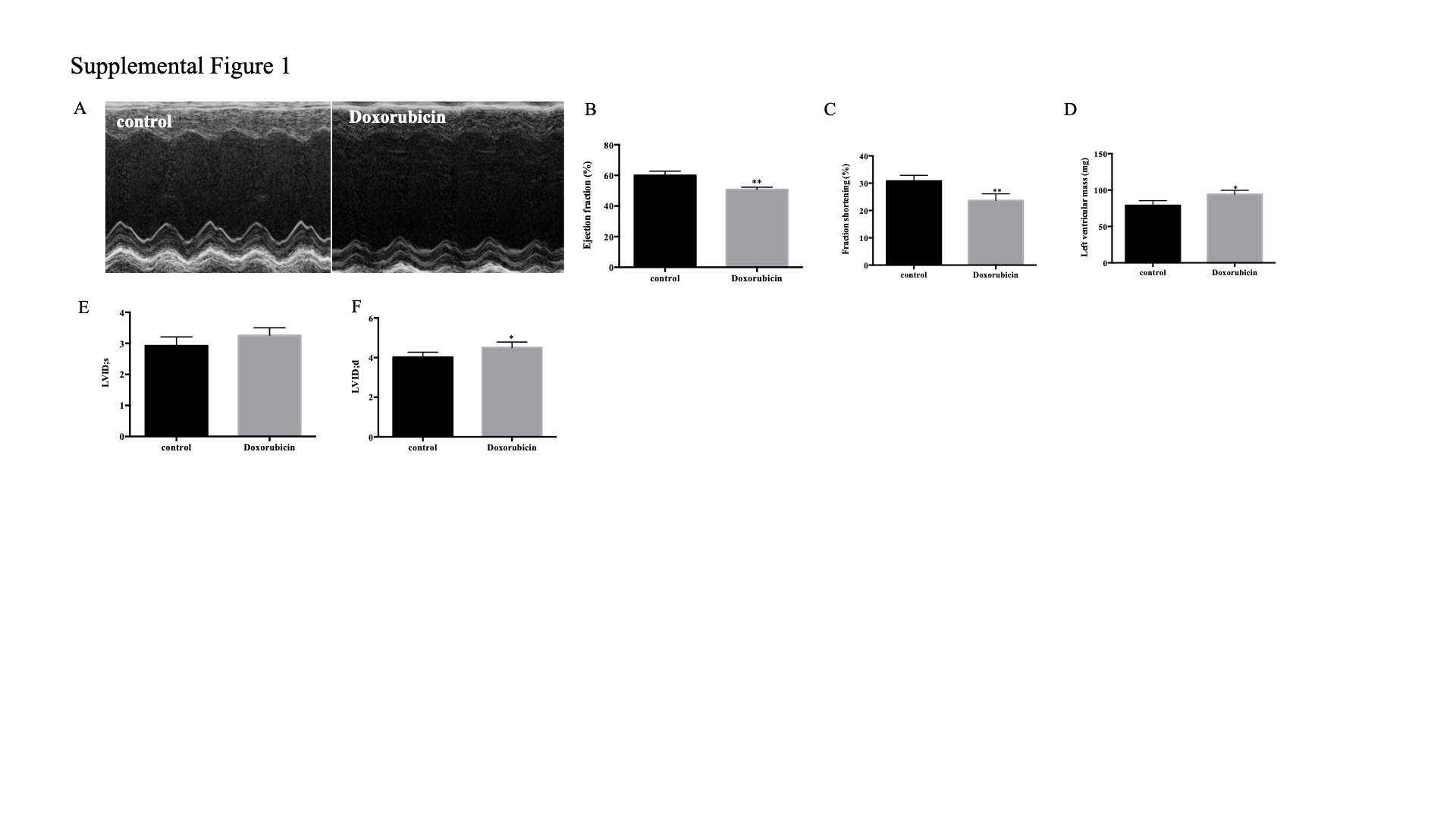

Supplement: Supplemental Figure 1 — The echocardiographic parameters including ejection fraction (EF), fraction shortenting (FS), left ventricular mass, left ventricular internal dimension-systole (LVIDs) and left ventricular internal dimension-diastole (LVIDd) for the doxorubicin-induced DCM model. *, p < 0.05;**, p < 0.01; ***, p < 0.001. [file Image_1.tiff]
